# The predictive and prognostic role of a novel ADS score in esophageal squamous cell carcinoma patients undergoing esophagectomy

**DOI:** 10.1186/s12935-018-0648-2

**Published:** 2018-10-03

**Authors:** Qiu-Fang Gao, Jia-Cong Qiu, Xiao-Hong Huang, Yan-Mei Xu, Shu-Qi Li, Fan Sun, Jing Zhang, Wei-Ming Yang, Qing-Hua Min, Yu-Huan Jiang, Qing-Gen Chen, Lei Zhang, Xiao-Zhong Wang, Hou-Qun Ying

**Affiliations:** Department of Clinical Laboratory, Jiangxi Province Key Laboratory of Laboratory Medicine, The Second Affiliated Hospital of Nanchang University, Nanchang, 330006 Jiangxi China

**Keywords:** Esophageal squamous cell cancer, Inflammation, ADS, Prognosis

## Abstract

**Background:**

Chronic inflammation is deemed to play a significant effect on initiation and progression of esophageal squamous cell carcinoma (ESCC). In current study, we investigated the prognostic and predictive role of albumin (Alb) to fibrinogen (Fib) ratio (AFR) and a novel AFR–Alb-derived neutrophil/lymphocyte ratio (dNLR) score (ADS) in ESCC patients undergoing esophagectomy and compared them with Fib, Alb, neutrophil to lymphocyte ratio (NLR), dNLR, platelet to lymphocyte ratio (PLR) and lymphocyte to monocyte ratio (LMR).

**Materials and methods:**

A total of 153 clinical confirmed ESCC patients undergoing esophagectomy between January 2011 and December 2013 were included in present study. We detected preoperative Alb, Fib and neutrophil, monocyte, lymphocyte and platelet count, and obtained overall survival (OS) by 3 years’ follow-up in the cases. X-tile software, Kaplan–Meier curve, Cox regression and predicted nomogram were used to evaluate the predictive and prognostic role of them in ESCC patients.

**Results:**

The optimal cut-off values of Fib, Alb, AFR, NLR, dNLR, PLR and LMR were 3.2 mg/dL, 38.2 g/L, 9.3, 2.1, 4.3, 145.9 and 2.3, respectively. High levels of Fib [(adjusted hazard ratio (HR) = 2.148, 95% confidential interval (CI) (1.229–3.753)], dNLR (adjusted HR = 2.338, 95% CI 1.626–5.308) and PLR (adjusted HR = 1.964, 95% CI 1.129–3.415) as well as low AFR (adjusted HR = 2.381, 95% CI 1.152–4.926) and Alb (adjusted HR = 2.398, 95% CI 1.342–4.273) were significantly associated with decreased OS in ESCC patients. The survival predictive areas under the time-dependent receiver operating characteristics curve of AFR, dNLR and Alb were higher than Fib and PLR, respectively. High ADS score was significantly associated with short 3 years’ OS of ESCC patients (adjusted HR = 2.94, 95% CI 1.70–5.08). Moreover, OS of ESCC patients receiving adjuvant radio-chemotherapy was longer than those without the treatment in high ADS score subgroup (*p *= 0.001), however, no significant survival difference was observed in the patients with or without treatment radio-chemotherapy (*p *= 0.297). Additionally, a significant difference was observed in c-index values of the nomograms including or without ADS (0.720 vs. 0.670, *p *< 0.05).

**Conclusions:**

Preoperative ADS was a prospective biomarker to predict clinical efficacy of adjuvant radio-chemotherapy and clinical prognosis of ESCC patients undergoing esophagectomy, and the score could apparently improve predicted efficacy of the nomogram.

**Electronic supplementary material:**

The online version of this article (10.1186/s12935-018-0648-2) contains supplementary material, which is available to authorized users.

## Background

Esophageal cancer is estimated to be the sixth common digestive malignancy and the fourth leading cause of cancer-related death in USA in 2017 [1]. In China, esophageal squamous cell carcinoma (ESCC) is the main kind of esophageal cancer, the incidence increases gradually in recent decade [2], and approximately 80% of the patients are diagnosed as the advanced disease with a poor prognosis [3]. Therefore, it is urgent to explore effective, economical and practical biomarker to diagnosis and predict recurrence and survival of the patients.

Cancer-related inflammation has been identified as one of the dominant features of ESCC [4]. It plays a significant effect on onset and metastasis of the disease [5–7]. Inflammation-related immune cell and acute phase reactive protein are the biomarkers of systematic inflammation, and the levels of them can reflect the degree of chronic inflammation in the patient. Preoperative neutrophil to lymphocyte ratio (NLR), derived neutrophil to lymphocyte ratio (dNLR), platelet to lymphocyte ratio (PLR) and lymphocyte to monocyte ratio (LMR) were reported to associate with clinical outcome in various malignancies, including ESCC [8–10]. However, it remains unclear the association of preoperative circulating fibrinogen (Fib), albumin (Alb) and Alb/Fib ratio (AFR) with survival of ESCC.

Fib is one of the vital elements in coagulation cascade, and hypercoagulation is commonly occurred in solid malignancies [11–13]. High level of circulating Fib was associated with poor survival of ESCC, lung cancer, hepatocellular carcinoma, respectively [14, 15]. Meanwhile, hypoalbuminemia was reported to detect in ESCC patients, and it was significantly associated with pathological stage of the patients [16, 17]. Thus, we speculate that AFR may be superior to the single Fib and Alb to predict progression and prognosis of ESCC.

In current study, we investigated the prognostic and predictive role of prospective AFR, Fib and Alb in 153 ESCC patients undergoing esophagectomy and compared their survival predicted efficacy with NLR, dNLR, PLR and LMR. Here, we reported a novel AFR–Alb–dNLR score (ADS) could independently predict survival of the surgical patient and precisely identify the cases who could benefit from adjuvant radio-chemotherapy.

## Materials and methods

In our study, we collected the eligible ESCC patients according to the following inclusion and exclusion criteria. Firstly, all ESCC patients were newly diagnosed and confirmed by histopathological examination in accordance with the 7th edition of the TNM-UICC/AJCC classification; secondary, all included patients underwent a total or subtotal esophagectomy with resection of at least 12 regional lymph nodes; thirdly, all enrolled patients were firstly diagnosed and didn’t received any other treatment before esophagectomy; fourth, follow-up data could obtained from all eligible patients. On the contrary, the patients with acute or chronic infection, autoimmune, hematological, liver disease or other malignancies as well as without clinical characteristics or survival data were excluded in our study. The study was approved by Medical Ethics Committee of the hospital, and all informed consents were signed and obtained from all included individuals.

The baseline demographic characteristics and pathological results were extracted from medical record. All the peripheral blood, plasma and serum samples were collected between 7:30 and 9:30 a.m. within 3 days before surgery. Clauss and bromocresol green methods were selected to detect plasma Fib and serum Alb using SYSMEX CA-7000 machine (Sysmex, Tokyo, Japan) and OLYMPUS AU5400 machine (Beckman Coulter, Tokyo, Japan), respectively. The inter- and intra-batch coefficient of variations of the two kits were less than 4.41% and 3.66%, 3.17% and 1.83%, respectively. Preoperative NLR, dNLR, PLR, LMR and AFR were calculated based on the laboratory detection. We carried out 3 years’ follow-up (3 months a time in the 1st and 2nd year, and 6 months in the 3rd year) by means of retrieving the medical record and telephone, and the deadline was December 2016. The time from surgery to death or the deadline was considered as overall survival (OS), and OS was the dominant endpoint of the study.

**Fig. 1:**
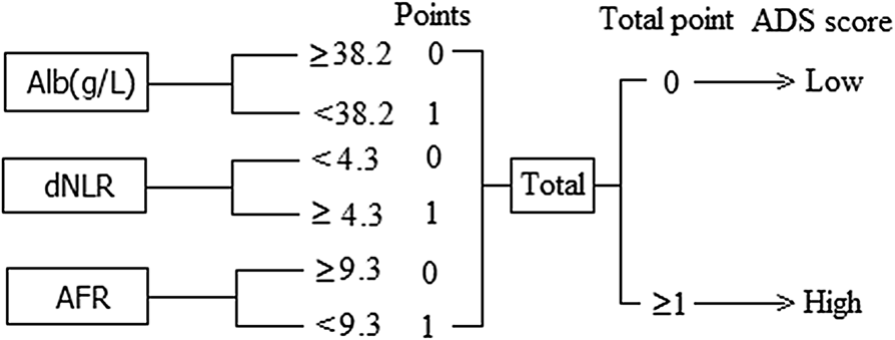
The detail definition of ADS score in present study

A novel inflammation-based prognostic score, ADS, was established in our study. Levels of Alb, AFR and dNLR which was higher or lower than the cut-off values were considered as 0 and 1 point, respectively. The total points with 0 and ≥ 1 were defined as low and high ADS score, respectively (Fig. 1).

Chi square test, Mann–Whitney *U* and Kruskal–Wallis test were used to investigate the relationships between clinical pathologic characteristics and these inflammatory biomarkers. X-tile software was selected to determine the optimal cut-off values of all inflammatory biomarkers. The sample power of present study was evaluated using PASS version 11.0.10 program (NCSS, California, USA). Kaplan–Meier survival curve (log-rank test) and Cox regression model were used to identify the independent prognostic factor for the disease. Predicted efficacy of the independent prognostic factor was evaluated and compared using time-dependent receiver operative characteristics curve (ROC). Predicted prognostic nomograms were constructed using the independent prognostic factors, and Harrell’s concordance index (c-index) was used to compare the difference between them. *p *< 0.05 was considered as the statistical difference in all statistics. All statistical analyses were conducted using SPSS 19.0 software (IBM Corp, Armonk, NY, USA), Graph Pad Prism 6 software (Inc, La Jolla, CA, USA), State 12 software (STATA Corp., College Station, TX, USA) and R 3.0.3 software (Institute for Statistics and Mathematics, Vienna, Austria).

## Results

In our study, a total of 153 ESCC patients treated with esophagectomy were recruited in our prospective study, and the sample power reached 78.8% when the determined hazard ratio was two at 0.05 significance level. All of the eligible patients were clinical confirmed and received esophagectomy with resection of at least 12 regional lymph nodes between January 2011 and December 2013 at the Second Affiliated Hospital of Nanchang University (Jiangxi, China). The baseline characteristics and laboratory results were summarized in Table 1. As shown from Table 1, 83.766% of the patients were male, and the average age was 61.93 ± 6.72 years. Seventy-nine and 74 of the patients were confirmed as TNM 0–II and III stage, and 80.4% and 19.6% of the cases showed well-moderate and poor cell differentiation. Sixty point one percent of the patients received adjuvant radio-chemotherapy. Numbers of the patient harbored low and high ADS score were 87 and 66, respectively. After follow-up, 93 (60.8%) patients were dead, and the median OS was 17.67 months.

**Table 1:** Clinical characteristics in 153 esophageal squamous cell carcinoma patients

Variables	Categories	No. of patients (%)
Gender	Male	128 (83.66)
	Female	25 (16.34)
Age	Year	61.93 ± 6.72
Tobacco	Yes	66 (43.14)
	No	87 (56.86)
Alcohol	Yes	57 (37.25)
	No	96 (62.75)
Hypertension	Yes	13 (8.50)
	No	140 (91.50)
Diabetes	Yes	2 (1.31)
	No	151 (98.69)
Tumor stage	0–II	79 (51.63)
	III	74 (48.37)
Depth of invasion	T1–T2	38 (24.84)
	T3	115 (75.16)
Lymph node	N0	79 (51.63)
	N1–N3	74 (48.37)
Differentiation	Well-moderate	123 (80.39)
	Poor	30 (19.61)
Tumor size (cm)	≤ 4	109 (71.24)
	> 4	44 (28.76)
Radio-chemotherapy	Yes	92 (60.13)
	No	61 (39.87)
NLR		2.88 (0.16–14.40)
dNLR		3.52 (0.01–24.07)
PLR		137.47 (8.38–673.68)
LMR		5.17 (1.03–65.40)
Fib	mg/dL	3.29 (1.8–5.42)
Alb	g/L	40.4 (22.8–49.8)
AFR		12.13 (5.74–22.15)
ADS	Low	87 (56.86)
	High	66 (43.14)

*NLR* neutrophil to lymphocytes ratio, *dNLR* derived neutrophil to lymphocyte ratio, *PLR* platelet to lymphocyte ratio, *LMR* lymphocyte to monocyte ratio, *Fib* fibrinogen, *Alb* albumin, *AFR* albumin to fibrinogen ratio, *ADS* AFR–Alb–dNLR score

**Fig. 2:**
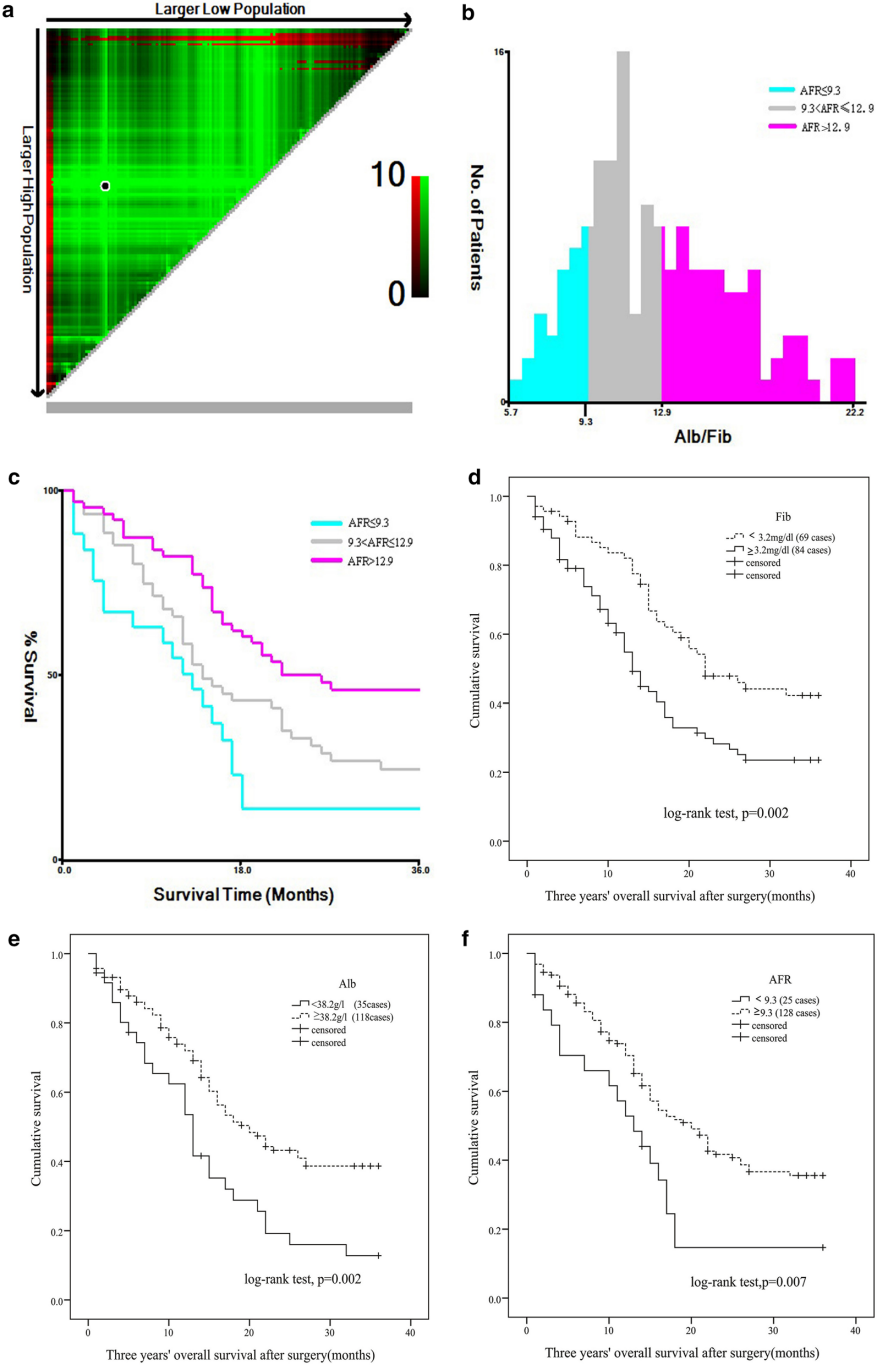
Association of preoperative AFR, Fib and Alb with progression and survival of esophageal squamous cell carcinoma. **a** 3Pop X-tile plot of AFR using X-tile software; **b** histogram of AFR using X-title software; **c** Kaplan–Meier curve of AFR using X-tile software. **d** Kaplan–Meier curve of Fib; **e** Kaplan–Meier curve of Alb; **f** Kaplan–Meier curve of AFR

**Fig. 3:**
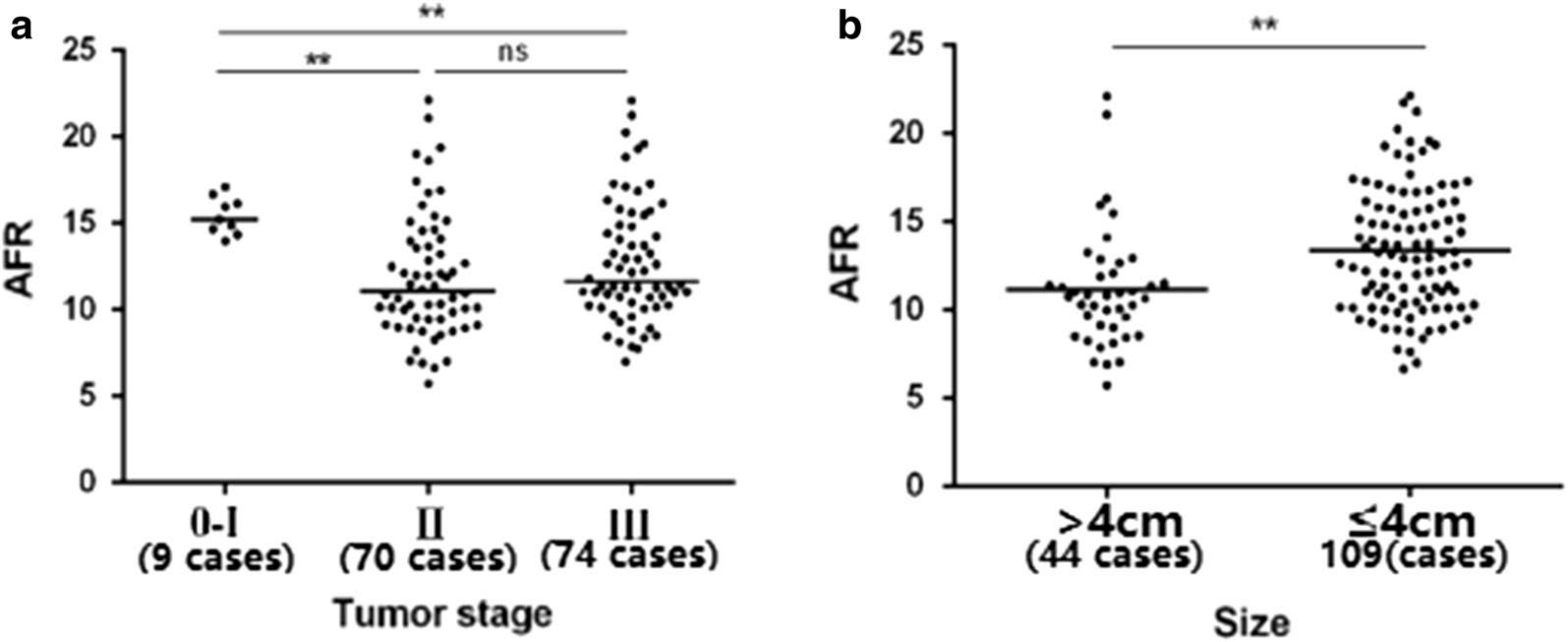
Association between AFR and TNM stage and tumor size in 153 eligible patients. **a** AFR in TNM 0–I, II and III stage subgroups; **b** AFR in different tumor size subgroups. *p < 0.05, **p < 0.01, ***p < 0.001, *ns* not significant

The optimal cut-off points of circulating NLR, dNLR, PLR, LMR, AFR, Fib and Alb were 2.1, 4.3, 145.9, 2.3, 9.3, 3.2 mg/dL and 38.2 g/L, respectively (Fig. 2a–c and Additional file 1: Figure S1). The cases were divided into high and low subgroups according to the cut-off points (Additional file 2: Table S1). Preoperative AFR was significantly associated with tumor size and stage as well as gender (Fig. 3), NLR was only positively associated with tumor size. However, no significant relationship was examined between age, alcohol, tobacco, hypertension, diabetes, depth of invasion and lymph node metastasis and the other inflammatory biomarkers.

**Fig. 4:**
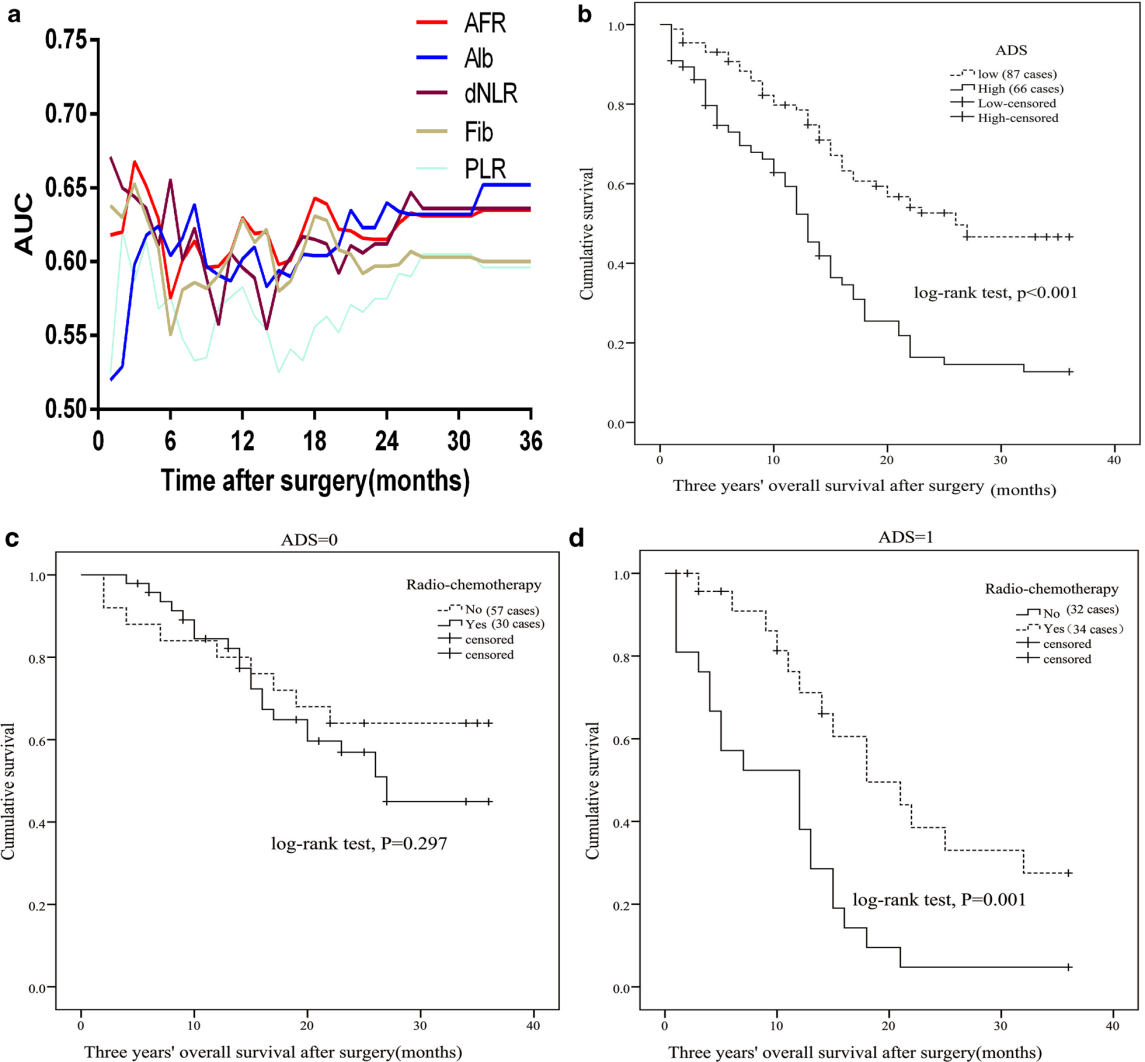
The prognostic role of preoperative AFR, Fib, Alb, dNLR, PLR and ADS in 153 patients with esophageal squamous cell carcinoma and Kaplan–Meier curve of radio-chemotherapy in subgroup stratified by ADS (high and low ADS score). **a** Time-dependent receiver operative characteristics curve of preoperative Fib, Alb, dNLR, AFR and PLR; **b** Kaplan–Meier curve of ADS; **c** Kaplan–Meier curve of radio-chemotherapy in low ADS subgroup; **d** Kaplan–Meier curve of radio-chemotherapy in high ADS subgroup

In the current study, the associations between clinical features, inflammatory biomarkers and clinical outcome of the cases were investigated. Poor differentiation (*p *= 0.047, crude HR = 2.041, 95% CI 1.008–4.131), invasive depth (T3) (*p *= 0.003, crude HR = 2.523, 95% CI 1.366–4.661), high levels of PLR (*p *= 0.019, crude HR = 1.64, 95% CI 1.085–2.479), dNLR (*p *< 0.001, crude HR = 2.387, 95% CI 1.515–3.761), Fib (*p *= 0.003, crude HR = 1.886, 95% CI 1.242–2.866) and low levels of LMR (*p *= 0.013, crude HR = 2.066, 95% CI 1.166–3.663), Alb (*p *= 0.003, crude HR = 1.969, 95% CI 1.266–3.058) and AFR (*p *= 0.01, crude HR = 1.953, 95% CI 1.175–3.247) were significantly associated with an increased risk of death for ESCC patients (Fig. 2d–f, Table 2 and Additional file 3: Figure S2). Moreover, invasive depth (*p *= 0.002, adjusted HR = 3.622, 95% CI 1.591–8.242), PLR (*p *= 0.017, adjusted HR = 1.964, 95% CI 1.129–3.415), dNLR (*p *< 0.001, adjusted HR = 2.338, 95% CI 1.626–5.308), Fib (*p *= 0.007, adjusted HR = 2.148, 95% CI 1.229–3.753), Alb (*p *= 0.003, adjusted HR = 2.398, 95% CI 1.342–4.273) and AFR (*p *= 0.019, adjusted HR = 2.381, 95% CI 1.152–4.926) were still significantly associated with the poor survival of the disease when adjusting by other variables (Table 2). Basing on the independent factors, the areas under time-dependent receiver operative characteristics curve (AUCs) of AFR, Alb and dNLR were higher than PLR and Fib (Fig. 4a).

**Table 2:** Univariate and multivariate Cox regression of clinical characteristics and inflammatory biomarkers in 153 esophageal squamous cell carcinoma patients

Variables	Three years’ OS			
	Univariate analysis		Multivariate analysis	
	HR (95% CI)	*p*-value	Adjusted HR (95% CI)^a^	*p*-value
Gender (male)	1.787 (0.927–3.445)	0.083	–	–
Age (≥ 60 years)	1.017 (0.663–1.559)	0.940	–	–
Tobacoo (yes)	1.093 (0.727–1.643)	0.670	–	–
Alcohol (yes)	1.065 (0.703–1.614)	0.767	–	–
Hypertension (yes)	1.102 (0.482–2.522)	0.818	–	–
Diabetes (yes)	20.837 (0.042–10283.099)	0.337	–	–
Tumor stage (III)	1.460 (0.945–2.256)	0.088	–	–
Depth of invasion (T3)	2.523 (1.366–4.661)	0.003	3.622 (1.591–8.242)	0.002
Lympth node (yes)	1.086 (0.717–1.645)	0.098	–	–
Differentiation (poor)	2.041 (1.008–4.131)	0.047	1.579 (0.641–3.889)	0.321
Tumor size (≥ 4 cm)	1.413 (0.910–2.193)	0.124	–	–
Radio-chemotherapy (yes)	1.444 (0.812–2.566)	0.211	–	–
NLR (≥ 2.1)	1.495 (0.967–2.311)	0.070	–	–
PLR (≥ 145.9)	1.640 (1.085–2.479)	0.019	1.964 (1.129–3.415)	0.017
LMR (< 2.3)	2.066 (1.166–3.663)	0.013	1.445 (0.669–3.115)	0.349
dNLR (≥ 4.3)	2.387 (1.515–3.761)	< 0.001	2.338 (1.626–5.308)	< 0.001
Fib (≥ 3.2 mg/dL)	1.886 (1.242–2.866)	0.003	2.148 (1.229–3.753)	0.007
Alb (< 38.2 g/L)	1.969 (1.266–3.058)	0.003	2.398 (1.342–4.273)	0.003
AFR (< 9.3)	1.953 (1.175–3.247)	0.010	2.381 (1.152–4.926)	0.019

*OS* overall survival, *HR* hazard ratio, *CI* confidence interval, *NLR* neutrophil to lymphocytes ratio, *dNLR* derived neutrophil to lymphocyte ratio, *PLR* platelet to lymphocyte ratio, *LMR* lymphocyte to monocyte ratio, *Fib* fibrinogen, *Alb* albumin, *AFR* albumin to fibrinogen ratio

^a^Adjusted by sex, age, alcohol, tobacco, hypertension, diabetes, radio-chemotherapy, tumor size, tumor grade and tumor stage

According to the result of time-dependent ROC, we established a novel inflammation-based score ADS. High score of ADS was significantly associated with poor survival comparing to the patient with low ADS in Kaplan–Meier curve (*p *< 0.001) (Fig. 4b). Result of Cox regression showed that the patient harbored the high score (crude HR = 2.59, 95% CI 1.71–3.92; adjusted HR = 2.94, 95% CI 1.70–5.08) was obviously associated with poor OS (Table 3).

**Table 3:** Univariate and multivariate Cox regression of ADS in 153 esophageal squamous cell carcinoma patients

Variable	Score	Number of patient	Three years’ OS			
			Univariate analysis		Multivariate analysis	
			HR (95% CI)	*p*-value	Adjusted HR (95% CI)^a^	*p*-value
ADS score	Low	87	1 (–)	–	1 (–)	–
	High	66	2.59 (1.71–3.92)	< 0.001	2.94 (1.70–5.08)	< 0.001

*OS* overall survival, *HR* hazard ratio, *CI* confidence interval, *ADS* AFR–Alb–dNLR score

^a^Adjusted by sex, age, alcohol, tobacco, hypertension, diabetes, radio-chemotherapy, tumor size, tumor grade and tumor stage

We compared OS of the patients receiving or without adjuvant radio-chemotherapy in subgroups of low and high ADS score. There was no survival difference in low ADS patient receiving or without radio-chemotherapy (*p *= 0.297). However, prognosis of the case receiving radio-chemotherapy was extremely longer than the case without the treatment in high ADS subgroup (*p *= 0.001) (Fig. 4c, d).

**Fig. 5:**
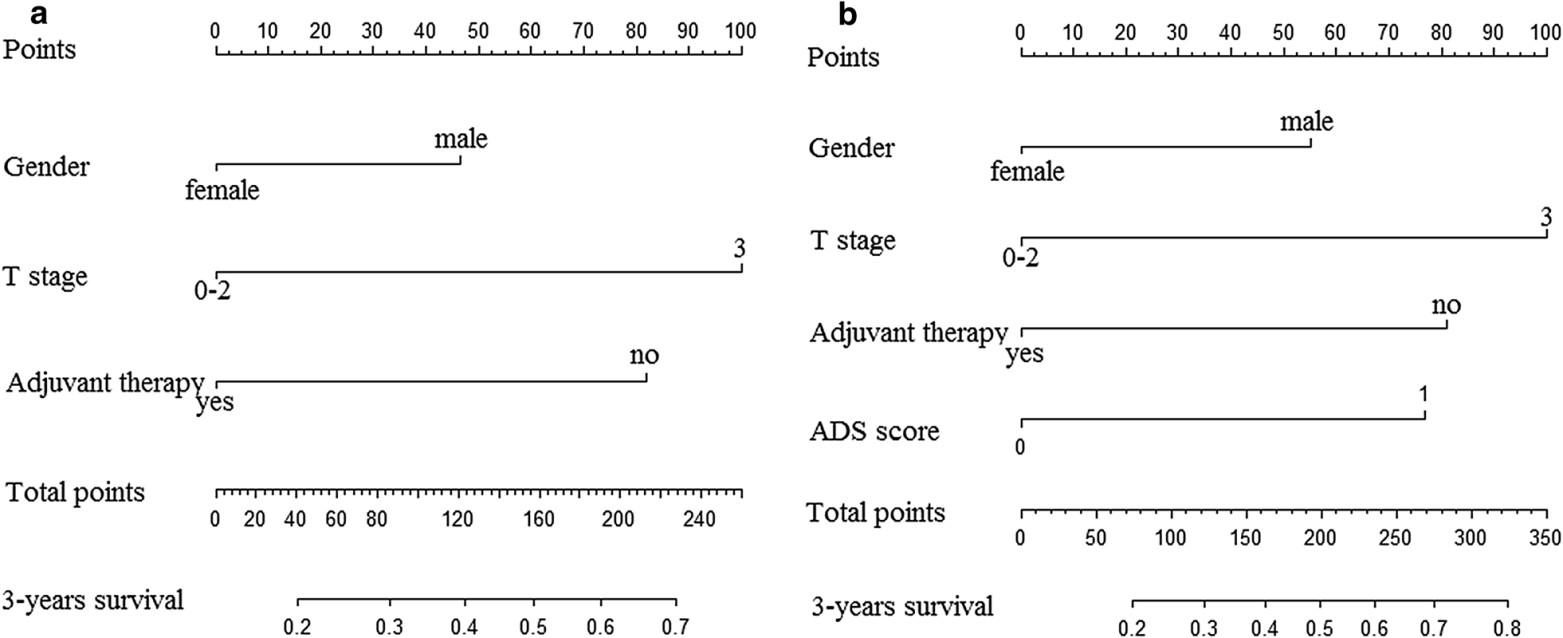
Predicted nomogram established by significant factors. **a** Without ADS score; **b** with ADS score

In order to further investigate the prognostic value of ADS score, the prognostic nomograms were established and all of them were listed in Fig. 5. The respective c-indexes of nomogram with or without ADS score were 0.720 and 0.670, and the significant difference was observed between them (*p *< 0.05).

## Discussion

ESCC was recognized as a complex disease causing by interaction of personal genetic susceptibility and environmental factor such as substantial intakes of alcohol and tobacco as well as frequent consumption of extremely hot beverage [18]. A persistent chronic inflammatory response triggered by these environmental exposures in the patients contributed to constitutive activation of pro-inflammatory signaling pathways, and to promote mutation of *P53*, *PIK3CA*, *RB1* and *NFE2L2*, consequently leading to onset and metastasis of ESCC. Therefore, the biomarkers which can reflect the severity of chronic inflammation may the candidate diagnostic, predicted or prognostic factors for the disease.

Till now, several studies reported the association of inflammatory biomarker with clinical outcome of ESCC. However, the controversial results were observed between them [19–21]. In our study, we comprehensively investigated circulating inflammatory related cells, proteins, and ratios of them to determine the simple, economical, practical clinical biomarker to precisely stratify the suitable patients to receive radio-chemotherapy and to predict the survival of ESCC. We found that preoperative AFR was obviously associated with tumor stage and size of ESCC, indicating that it could predict progression and cancer burden of the disease. Obvious positive associations were observed between AFR, ADS score, dNLR, PLR, LMR and survival of the patient, and the adjusted HR of ADS score was larger than those of AFR, dNLR, PLR and LMR, respectively, suggesting that the score was superior to these single biomarkers to predict the survival of the patients. The predicted efficacy of nomogram including ADS score was higher than that without it, illustrated that this score could improve the predicted accuracy of ESCC nomogram. Clinical survival of the patients with treatment of adjuvant radio-chemotherapy was longer than the cases without the therapy only in high ADS subgroup, demonstrating that only the patient harbored high ADS score could benefit from adjuvant radio-chemotherapy.

ESCC is a chronic consumption disease with systematic inflammation, and most of the patients are commonly observed to be emaciated with restricted oral intake [22]. Moreover, the lower Alb implys worse nutritional status. For this, decreased Alb was significantly associated with poor survival in our study. Cancer associated fibroblast and tumor cell could secreted interleukin-6 to inhibit and stimulate Alb and Fib, respectively [23]. Meanwhile, Fib was also observed to bind with fibroblast growth factor-2 and vascular endothelial growth factor as well as platelet-derived growth factor with high affinity. The binding complex could induce epithelial–mesenchymal transition to promote invasion and metastasis of ESCC cell [24]. Moverover, hyperfibrinogenemia was observed not only in our study, but also reported by Suzuki et al. [25] and Kijima et al. [26], and preoperative circulating Fib was an independent prognostic factor for ESCC [27]. Hence, we observed that AFR was superior to Fib to predict the survival of the ESCC patients. In ESCC microenvironment, significant low Foxp3+, CD4+ and CD8+ T cells were observed to correlate with improved survival of the cancer [28], and high tumor-infiltrating neutrophil was significantly associated with progression of the disease [29]. Furthermore, an elevated dNLR was reported to be an independent prognostic biomarker for clinical outcome of the patient with treatment of definitive chemoradiotherapy [30]. In accordance with the above reasons, we found that ADS score based on Alb, AFR and dNLR was superior to the single biomarker to precisely predict cancer burden and prognosis of the disease.

This study is the first time for us to establish ADS score and to investigate the predictive and prognostic roles of it in ESCC patients undergoing esophagectomy. Moreover, the score was superior to AFR to predict the survival of ESCC, avoiding false positive or negative result. Additionally, the nomogram was a convenient and efficient tool for both the patients and doctors to choose the suit treatment and to predict the prognosis. However, several limitations of our study should be addressed as following. Firstly, only 153 eligible patients were included in our study, since small sample size could lead to unstable result. Secondary, although the study was a prospective design, all of the included patients were included from single hospital, the conclusion wasn’t validated by other centers. Thirdly, only 3 years’ follow-up was performed in our study, we couldn’t obtain the sufficient recurrent/metastatic data and long follow-up from these patients, it remained unknown the association between AFR, ADS and recurrence or progression of surgical ESCC patients. For this, further large sample size and multi-center studies are warrant to confirm our results.

## Conclusions

In conclusion, preoperative ADS score was an effective and independent factor for predicting prognosis of surgical ESCC patients, and it could effectively distinguish the patients who could benefit from adjuvant radio-chemotherapy.

## Additional files

**Additional file 1: Figure S1.** The optimal cut-off of preoperative NLR, dNLR, PLR, LMR, Fib, Alb lever in 153 surgical esophageal squamous cell carcinoma patients using X-tile software. **A**: NLR; **B**: dNLR; **C**: PLR; **D**: LMR; **E**: Fib; **F**: Alb.

**Additional file 2: Table S1.** Correlation of preoperative Alb, AFR, NLR, dNLR and clinicopathological characteristics in 153 esophageal squamous cell carcinoma patients.

**Additional file 3: Figure S2.** Kaplan–Meier curve of dNLR, PLR, LMR, Fib, Alb, AFR in 153 surgical esophageal squamous cell carcinoma patients. **A**: dNLR; **B**: PLR; **C**: LMR; **D**: Fib; **E**: Alb; **F**: AFR.

## Abbreviations

ADS: AFR–Alb–dNLR score
ESCC: esophageal squamous cell carcinoma
Alb: albumin
Fib: fibrinogen
AFR: Alb/Fib ratio
dNLR: derived neutrophil/lymphocyte ratio
NLR: neutrophil/lymphocyte ratio
PLR: platelet/lymphocyte ratio
LMR: lymphocyte/monocyte ratio
OS: overall survival
HR: hazard ratio
95% CI: 95% confidential interval
ROC: receiver operative characteristics curve
c-index: Harrell’s Concordance Index
AUC: the areas under time-dependent receiver operative characteristics curve
