# Supplementary figures and images for: The predictive and prognostic role of a novel ADS score in esophageal squamous cell carcinoma patients undergoing esophagectomy
Source: Cancer Cell Int. 2018 Oct 3;18:153. doi: 10.1186/s12935-018-0648-2 (PMC6171189; doi:10.1186/s12935-018-0648-2)

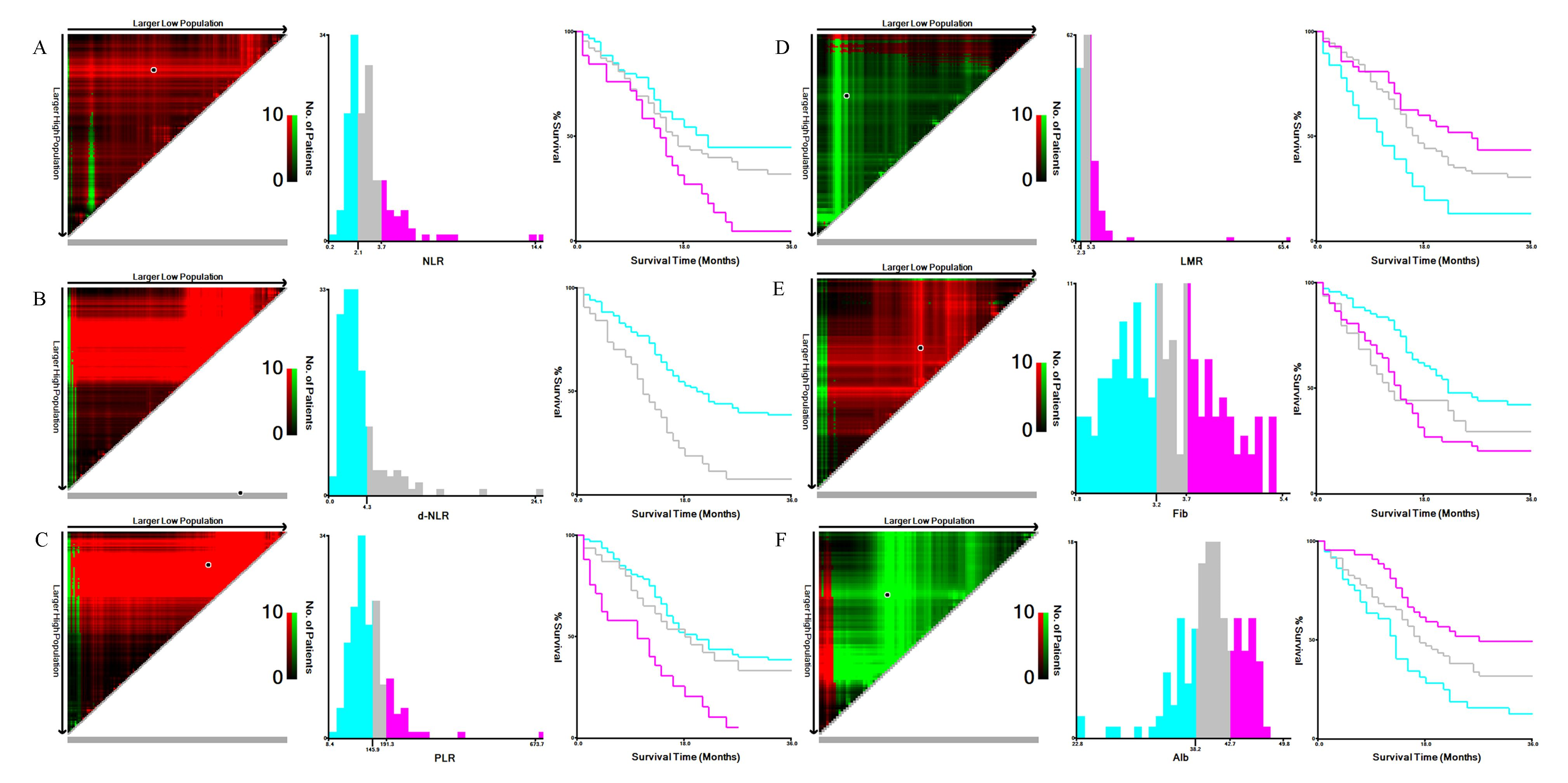

Supplement: Supplementary file 1 — Additional file 1: Figure S1. The optimal cut-off of preoperative NLR, dNLR, PLR, LMR, Fib, Alb lever in 153 surgical esophageal squamous cell carcinoma patients using X-tile software. A: NLR; B: dNLR; C: PLR; D: LMR; E: Fib; F: Alb. [file 12935_2018_648_MOESM1_ESM.tif]

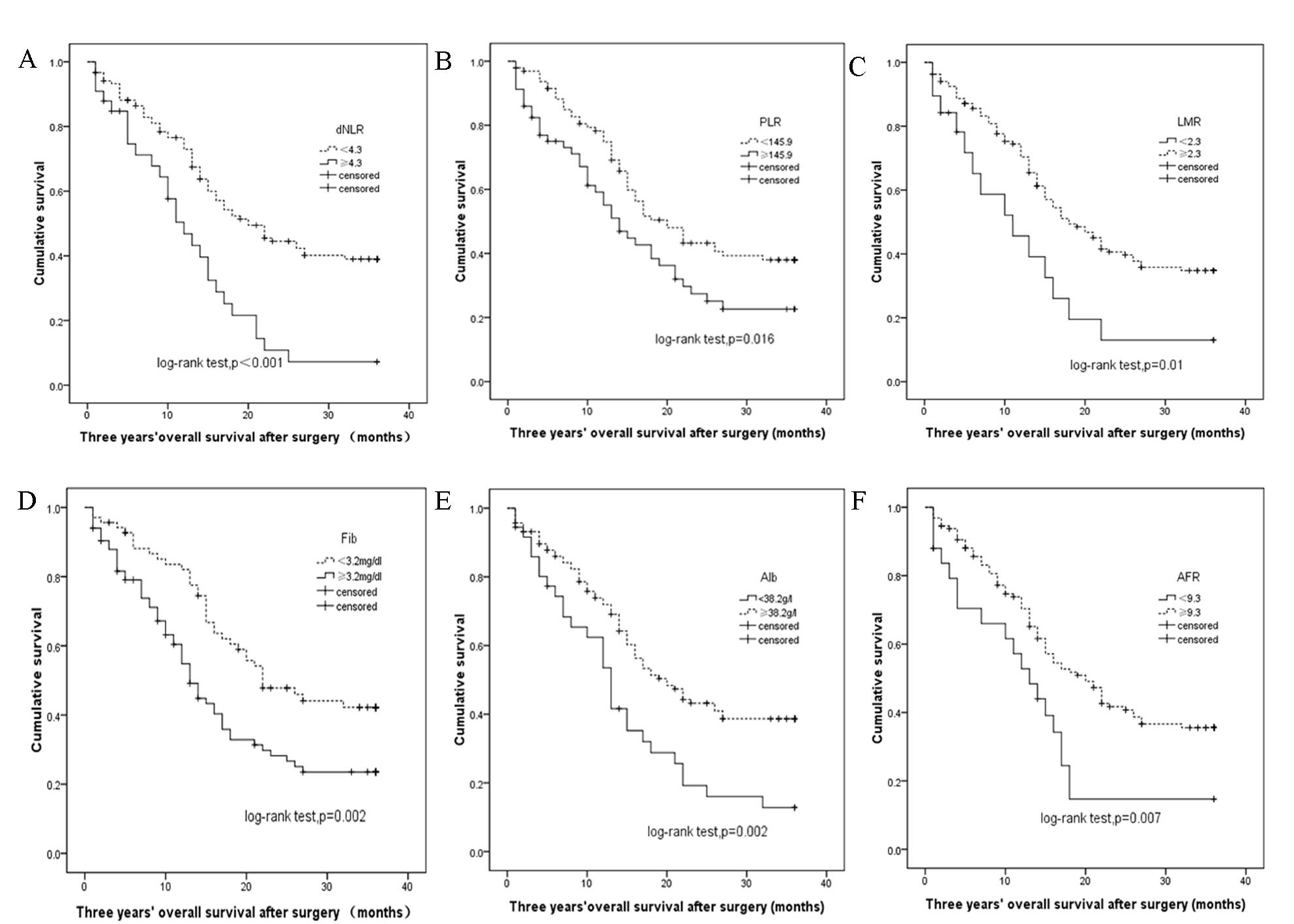

Supplement: Supplementary file 3 — Additional file 3: Figure S2. Kaplan–Meier curve of dNLR, PLR, LMR, Fib, Alb, AFR in 153 surgical esophageal squamous cell carcinoma patients. A: dNLR; B: PLR; C: LMR; D: Fib; E: Alb; F: AFR. [file 12935_2018_648_MOESM3_ESM.tif]
