# Supplementary material for: The predictive and prognostic role of a novel ADS score in esophageal squamous cell carcinoma patients undergoing esophagectomy
Source: Cancer Cell Int. 2018 Oct 3;18:153. doi: 10.1186/s12935-018-0648-2 (PMC6171189; doi:10.1186/s12935-018-0648-2)
Supplement: Supplementary file 2 — Additional file 2: Table S1. Correlation of preoperative Alb, AFR, NLR, dNLR and clinicopathological characteristics in 153 esophageal squamous cell carcinoma patients. [file 12935_2018_648_MOESM2_ESM.doc]

**Table S1:** Correlation of preoperative Alb, AFR, NLR, dNLR and clinicopathological characteristics in 153 esophageal squamous cell carcinoma patients.

| **Characteristics** | | **Alb subgroups ≥38.2 <38.2** | ***P*-value** | **AFR subgroups ≥9.3 <9.3** | ***P*-value** | **NLR subgroups ≥2.1 <2.1** | ***P*-value** | **dNLR subgroups ≥4.3 <4.3** | ***P*-value** |
| --- | --- | --- | --- | --- | --- | --- | --- | --- | --- |
| Gender | Male | 99 29 | 0.565 | 104 24 | 0.049 | 83 45 | 0.112 | 25 103 | 0.166 |
|  | Female | 18 7 |  | 24 1 |  | 12 13 |  | 8 17 |  |
| Age(years) | <60 | 41 11 | 0.619 | 43 9 | 0.816 | 34 18 | 0.547 | 9 43 | 0.358 |
|  | ≥60 | 76 25 |  | 85 16 |  | 61 40 |  | 24 77 |  |
| Tobacoo | Yes | 52 14 | 0.556 | 57 9 | 0.431 | 37 29 | 0.18 | 13 53 | 0.624 |
|  | No | 65 22 |  | 71 16 |  | 58 29 |  | 20 67 |  |
| Alcohol | Yes | 45 12 | 0.578 | 49 8 | 0.552 | 36 21 | 0.834 | 11 46 | 0.599 |
|  | No | 72 24 |  | 79 17 |  | 59 37 |  | 22 74 |  |
| Hypertension | Yes | 9 4 | 0.506 | 11 2 | 1 | 9 4 | 0.56 | 3 10 | 1 |
|  | No | 108 32 |  | 117 23 |  | 86 66 |  | 30 110 |  |
| Diabetes | Yes | 2 0 | 1 | 2 0 | 1 | 1 1 | 1 | 0 2 | 1 |
|  | No | 115 36 |  | 126 25 |  | 94 57 |  | 33 118 |  |
| Tumor stage | 0-II | 60 19 | 0.816 | 62 17 | 0.194 | 48 31 | 0.995 | 17 62 | 0.794 |
|  | III | 55 19 |  | 64 10 |  | 45 29 |  | 17 56 |  |
| Depth of invasion | T1-T2 | 25 13 | 0.155 | 30 8 | 0.613 | 21 17 | 0.317 | 6 32 | 0.271 |
|  | T3 | 89 26 |  | 95 20 |  | 74 41 |  | 28 87 |  |
| Lymph node | N0 | 58 21 | 0.749 | 61 18 | 0.138 | 51 28 | 0.516 | 16 63 | 0.545 |
|  | N1-N3 | 56 18 |  | 64 10 |  | 44 30 |  | 18 56 |  |
| Differentiation | Well-moderate | 91 32 | 0.762 | 104 19 | 0.065 | 77 46 | 0.792 | 23 100 | 0.034 |
|  | Poor | 23 7 |  | 21 9 |  | 18 12 |  | 11 19 |  |
| Tumor size(cm) | ≤4 | 87 22 | 0.125 | 97 12 | 0.005 | 62 47 | 0.005 | 21 88 | 0.166 |
|  | >4 | 30 14 |  | 31 13 |  | 36 8 |  | 13 31 |  |
| Radio-chemotherapy | Yes | 77 15 | 0.085 | 81 11 | 0.615 | 55 37 | 0.925 | 18 74 | 0.614 |
|  | No | 44 17 |  | 52 9 |  | 36 25 |  | 14 47 |  |
| OS | Alive | 64 29 | 0.005 | 74 19 | 0.088 | 63 30 | 0.073 | 27 66 | 0.005 |
|  | Dead | 53 7 |  | 54 6 |  | 32 28 |  | 6 54 |  |

**Abbreviation:** NLR: neutrophil to lymphocytes ratio; dNLR: derived neutrophil to lymphocyte ratio; Alb: albumin; AFR: albumin to fibrinogen ratio.
